# Draft genome sequencing of a multidrug-resistant *Escherichia coli* strain MBBL25_EM1 isolated from a cow diagnosed with endometritis

**DOI:** 10.1128/mra.00928-25

**Published:** 2025-10-10

**Authors:** Mahmuda Nasrin Juthi, Tanvir Shahriar, Md. Morshedur Rahman, Kazi Khalid Ibne Khalil, Anup Kumar Talukder, M. Nazmul Hoque, Ziban Chandra Das

**Affiliations:** 1 Molecular Biology and Bioinformatics Laboratory, Department of Gynecology, Obstetrics and Reproductive Health, Gazipur Agricultural University (GAU) 198780, Gazipur, Bangladesh; 2 Department of Physiology and pharmacology, GAU 198780 , Gazipur, Bangladesh; University of Maryland School of Medicine, Baltimore, Maryland, USA

**Keywords:** *E. coli*, multidrug resistance, endometritis, cows, whole genome

## Abstract

This study reports the draft genome of a multidrug-resistant *Escherichia coli* MBBL25_EM1, isolated from a crossbred dairy cow with endometritis. The 4.8 Mbp genome, assembled into 139 contigs, harbors 50 antimicrobial resistance genes and 52 virulence factor genes, highlighting its pathogenic potential in causing endometritis in dairy cows.

## ANNOUNCEMENT

*Escherichia coli*, a ubiquitous gram-negative bacterial pathogen, is usually responsible for infection in various hosts, such as human and animal, and known as a causative agent for endometritis of dairy cows (1). *E. coli* is of great concern as it has the potential to acquire multidrug resistance and is also a zoonotic pathogen (1, 2). This study aims to report the draft genome sequence of an *E. coli* isolate, MBBL25_EM1, isolated from the uterine discharge of a crossbred dairy cow clinically diagnosed with endometritis in a dairy farm located at the Gazipur district (23.99°N, 90.41°E) of Bangladesh. A uterine sample was collected according to the standard veterinary procedure for postpartum cows (3). For the isolation of *E. coli*, swab samples were enriched overnight in nutrient broth (Oxoid, UK) at 37°C under aerobic conditions, followed by a 10⁻³ serial dilution and streaking onto Eosin Methylene Blue agar (Oxoid) (2, 4, 5). Colonies exhibiting a metallic green sheen after 24 h were sub-cultured, and the isolates were identified as *E. coli* based on colony morphology, Gram staining (gram-negative), and biochemical tests (2, 6). Species-level identification of *E. coli* was performed using the VITEK-2 System v.9.01 (7). MBBL25_EM1 was tested against 15 antibiotics and showed resistance to vancomycin, oxacillin, colistin sulfate, polymyxin B, and streptomycin, as determined by Kirby-Bauer disk diffusion following Clinical and Laboratory Standards Institute M100 (2023) guidelines (8). Genomic DNA was extracted from pure colonies of MBBL25_EM1 grown on nutrient agar at 37°C for 24 h using the QIAamp DNA Mini Kit (QIAGEN, Germany) (9, 10). Sequencing libraries were prepared from 1 ng of DNA with the Nextera XT Kit (Illumina, USA) and sequenced on an Illumina MiSeq platform with 2 × 250 bp paired-end reads (11, 12).

Raw reads (*n* = 3,977,952) underwent trimming with Trimmomatic v.0.39 (13) and quality checking through FastQC v.0.11.7 (14). The draft genome was assembled using SPAdes v.3.15.5 (15) and annotated using the National Center for Biotechnology Information Prokaryotic Genome Annotation Pipeline v.6.10 (16). Genome completeness, antimicrobial resistance genes (ARGs), virulence factor gene (VFGs), plasmid, pathogenicity score, and clustered regularly interspaced short palindromic repeats (CRISPR) arrays in the draft genome were predicted using CheckM v.1.2.4 (17), CARD v.4.0.1 (18), VFDB v.6.0 (19), PlasmidFinder v.2.0.1 (20), PathogenFinder2 v.0.5.0 (21), and CRISPRCasFinder v.2.0.3 (22), respectively. All the tools used in this study were used with default parameters unless otherwise stated.

**Fig 1:**
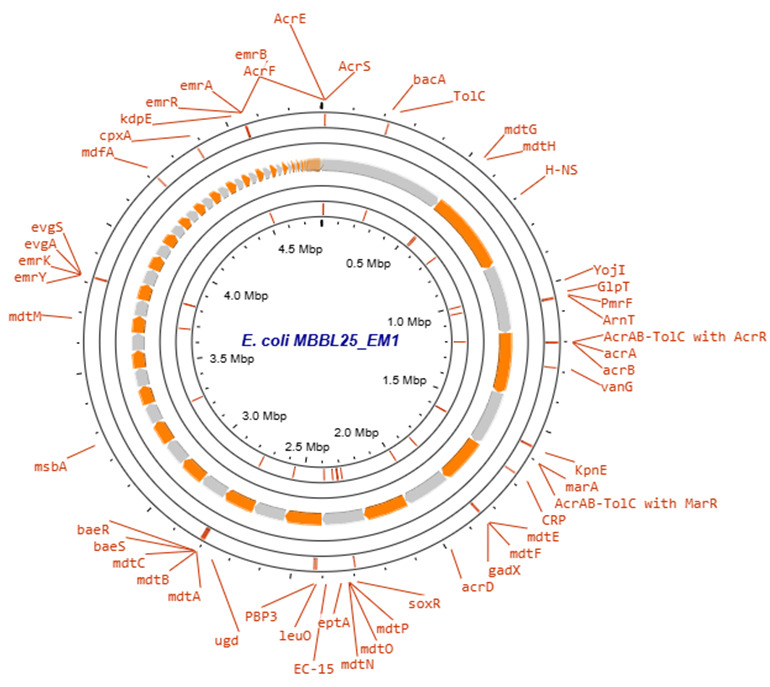
*E. coli* MBBL25_EM1 circular genome representing antibiotic resistance genes. The orange and gray arrows in the middle represent the open reading frames of the genome. The circular genomic map was created using the Proksee server (https://proksee.ca/).

The features of the draft genome are presented in Table 1 and Fig. 1. We predicted 50 ARGs, 52 VFGs (e.g., *gspJ*, *gtrA*, *espL1*, and *entB*), 2 CRISPR arrays, and 3 plasmid replicons in the draft genome. Additionally, PathogenFinder predicted *E. coli* MBBL25_EM1 as highly pathogenic, with a score of 0.946, and matches to 673 pathogenic families. These findings underscore the significant involvement of *E. coli* in clinical endometritis and provide insights into its AMR and virulence for endometritis management in dairy farms.

**TABLE 1:** Genomic features of *E. coli* strain MBBL25_EM1 isolated from uterine discharge with clinical endometritis^*a*^

Genome feature	*Escherichia coli* (MBBL25_EM1)
GenBank accession	JBPUSH000000000.1
Assembled genome size (bp)	4,804,213
GC (%)	50.7
Coverage (×)	103
Genome completeness (%)	99.56
CheckM contamination (%)	0.37
BioSample accession no.	SAMN49982472
BioProject accession no.	PRJNA1291960
*N*_50_ value (bp)	176,117
Number of contigs	139
Genes (total)	4,720
CDSs (total)	4,638
Genes (coding)	4,519
CDSs (with protein)	4,519
Genes (RNA)	82
rRNAs	3, 3, 1 (5S, 16S, 23S)
Complete rRNAs	1 (23S)
tRNAs	67
ncRNAs	8
Pseudogenes (total)	119
CDSs (without protein)	119
Pseudogenes (ambiguous residues)	0 of 119
Pseudogenes (frameshifted)	53 of 119
Pseudogenes (incomplete)	62 of 119
Pseudogenes (internal stop)	23 of 119
Pseudo genes (multiple problems)	18 of 119
CRISPR arrays	2
Number of ARGs	50
Number of VFGs	52
PathogenFinder score	0.946
MBBL25_EM1 matched with pathogenic families	673

^*a*^ CDS, coding sequence.

## Data Availability

The whole genome shotgun project of *Escherichia coli* strain MBBL25_EM1 has been deposited in GenBank and the National Center for Biotechnology Information Sequence Read Archive (SRA) (SRA accession number SRR34550966) under BioProject accession number PRJNA1291960. The version described in this paper is version JBPUSH000000000.1.
